# Bisecting Lewis X in Hybrid-Type *N*-Glycans of Human Brain Revealed by Deep Structural Glycomics

**DOI:** 10.1021/acs.analchem.1c03793

**Published:** 2021-11-01

**Authors:** Johannes Helm, Clemens Grünwald-Gruber, Andreas Thader, Jonathan Urteil, Johannes Führer, David Stenitzer, Daniel Maresch, Laura Neumann, Martin Pabst, Friedrich Altmann

**Affiliations:** Department of Chemistry, University of Natural Resources and Life Sciences Vienna, Muthgasse 18, 1190 Vienna, Austria

## Abstract

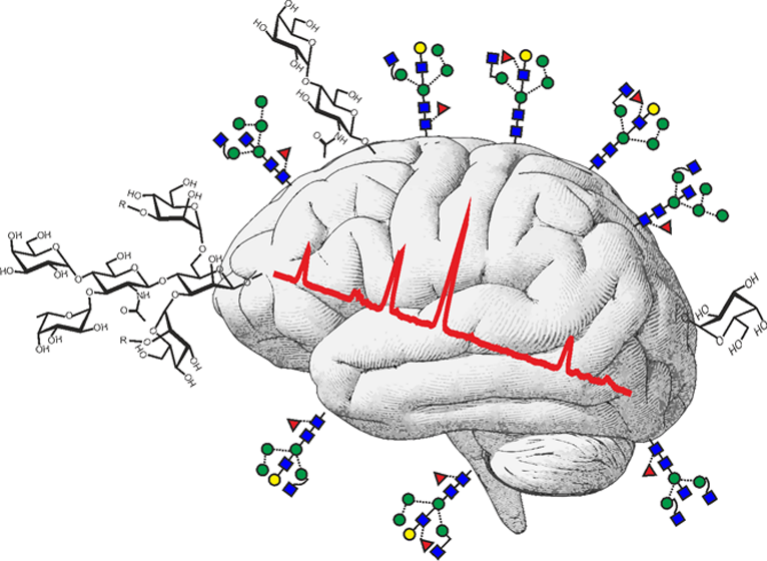

The importance of protein glycosylation in the biomedical field requires methods that not only quantitate structures by their monosaccharide composition, but also resolve and identify the many isomers expressed by mammalian cells. The art of unambiguous identification of isomeric structures in complex mixtures, however, did not yet catch up with the fast pace of advance of high-throughput glycomics. Here, we present a strategy for deducing structures with the help of a deci-minute accurate retention time library for porous graphitic carbon chromatography with mass spectrometric detection. We implemented the concept for the fundamental *N*-glycan type consisting of five hexoses, four *N*-acetylhexosamines and one fucose residue. Nearly all of the 40 biosynthetized isomers occupied unique elution positions. This result demonstrates the unique isomer selectivity of porous graphitic carbon. With the help of a rather tightly spaced grid of isotope-labeled internal *N*-glycan, standard retention times were transposed to a standard chromatogram. Application of this approach to animal and human brain *N*-glycans immediately identified the majority of structures as being of the bisected type. Most notably, it exposed hybrid-type glycans with galactosylated and even Lewis X containing bisected *N*-acetylglucosamine, which have not yet been discovered in a natural source. Thus, the time grid approach implemented herein facilitated discovery of the still missing pieces of the *N*-glycome in our most noble organ and suggests itself—in conjunction with collision induced dissociation—as a starting point for the overdue development of isomer-specific deep structural glycomics.

Individual glycoforms of glycoproteins confer biological properties and are increasingly considered as markers for health and disease.^1−5^ The introduction of fluorescent labels for liquid chromatography (LC) or capillary electrophoresis,^6−10^ the development of electrospray ionization-mass spectrometry (ESI-MS)^11^ and matrix-assisted time-of-flight MS (MALDI-MS) for native or derivatized glycans,^12^ and the versatility of LC coupled to ESI-MS for glycopeptides^13,14^ have brought glycan analysis into the “omics” era. The price for high throughput, however, all too often is an oversimplification of results by ignoring the possible, often simultaneous occurrence of a large number of isomers. Identification of structural isoforms of *N*-glycans is still one of the “grand challenges” in glycomics of complex samples,^15,16^ which arguably requires separation of isomers.^17^ Hydrophilic interaction (HILIC) HPLC with amide-functionalized stationary phases has found wide applications offering the advantage of identical molar response of all *N*-glycan species in a sample. Correct quantitation requires good separation of peaks, which is facilitated by ultrahigh-performance HILIC columns,^8,18^ which, however, reach their limit with triantennary glycans. Retention is based on number and—to a limited degree—on position of the hydroxyl groups of a glycan. Thus, HILIC-HPLC can just about distinguish between core and outer arm fucosylation.^19^ However, the retention time differences provided even by ultraperformance HILIC columns can hardly be seen as sufficient to discriminate over 40 isomers of the fundamental *N*-glycan composition of five hexoses, four *N*-acetyl-hexosamines, and one fucose (H5N4F1) (Figure 1). The recently introduced coupling of HILIC-HPLC and ESI-MS certainly improves the cognitive gain,^20^ but does not substantially increase the ability to separate and annotate *N*-glycan isomers.

**Figure 1:**
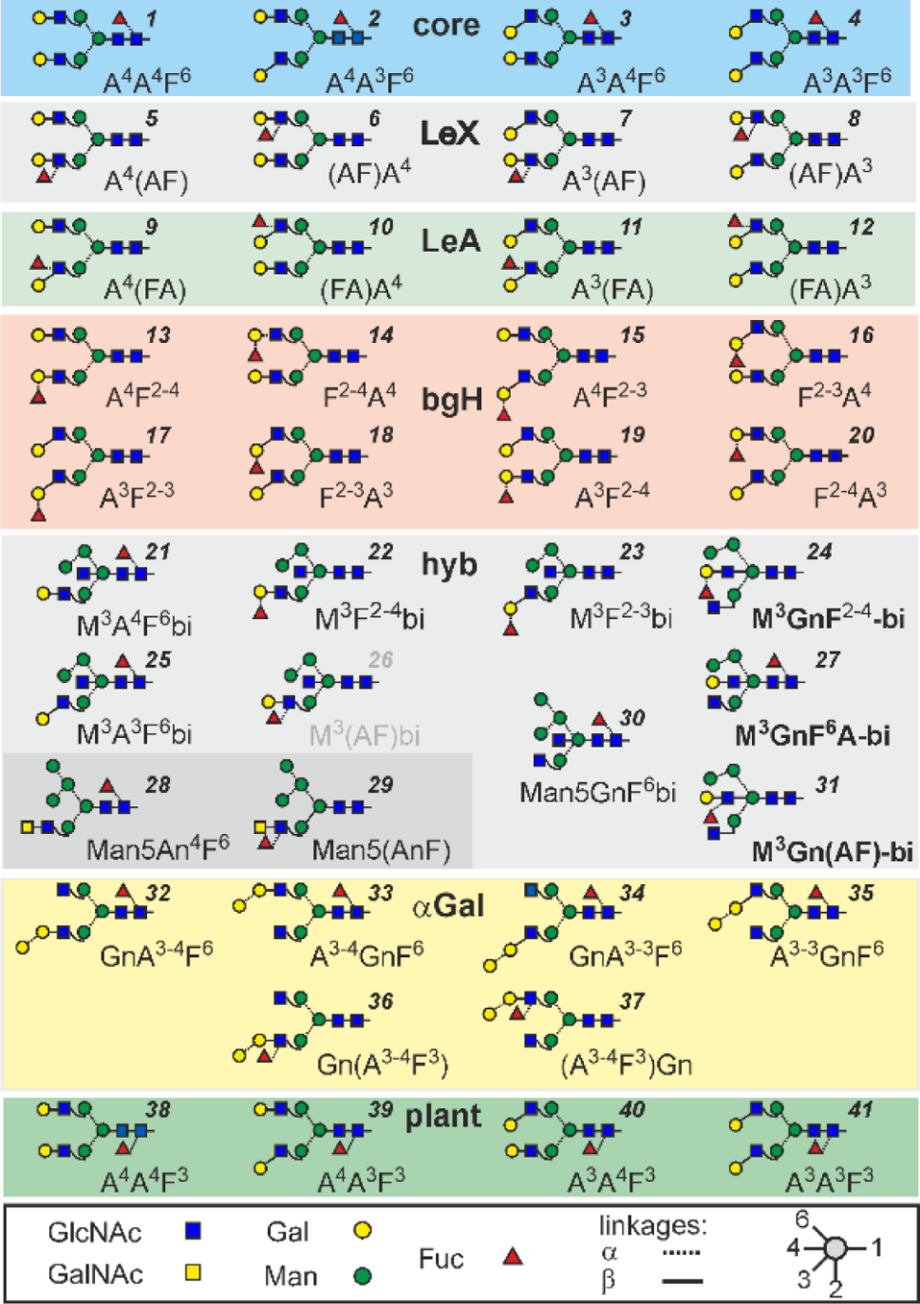
Fundamental *N*-glycan structures composed of five hexoses, four *N*-acetylhexosamines and one fucose (H5N4F1) used in this study. Names of supposedly novel structures are boldfaced. The abbreviation system is explained in the Supporting Information. Tentative numbers for quick referencing are given in italic print.

Much higher resolution of isomeric glycans is achieved by porous graphitized carbon (PGC) chromatography, which exerts its superb ability only with underivatized glycans and hence requires MS detection. Negative ion collision induced decay (CID) provides valuable hints at a glycan’s structure^21−25^ but reaches limits with linkage isomers. The utilization of retention times^21^ is hampered by poor reproducibility. This problem could be solved by isotope-labeled internal standards. Chemical synthesis has recently been employed to synthesize a range of ^13^C-labeled *N*-glycans.^26^ An elution time matching that of a certain glycan does, however, not rule out the possibility of this glycan actually being another isomer unless an incidental co-elution can be ruled out by CID^22−24^ or by knowing the retention times of all possible isomers. In the absence of such knowledge, three recent comprehensive glycomic studies of brain tissues admittedly had to refrain from assigning explicit structures.^22,27−29^ Nevertheless, these and other studies revealed an unusually high amount of bisecting GlcNAc in brains of various species.^22,27,29−32^

As of today, one can postulate that probably all human and mammalian glycosyltransferases and structural features are known and thus the glycome space, that is, the bona fide entirety of all isomeric *N*-glycan structures of a given mass level can be predicted.^33^ Establishing the relative retention times of all possible isomers, when separated by a shape-selective phase such as PGC,^21,34^ provides a rational approach for structural assignment. At least, the many isomers not fulfilling the retention criterium can be ruled out right away. First steps in this direction have been attempted with a selection of the most likely occurring disialo *N*-glycans^35^ and with oligomannosidic *N*-glycans.^36^

In the present work, we extend the range of synthetic reference structures with complex-type *N*-glycans containing fucose with its many attachment and interaction options, and we introduce a solution for overcoming retention time differences, which may derive from different columns, gradients, operators, or frequently observed “aging” (redox reactions) of PGC. Thirty-six isomers of glycans containing five hexoses, four *N*-acetylhexosamines and one fucose (H5N4F1), that may occur in mammals were biosynthetized. The PGC runs were conducted in the presence of eight synthetic (internal) standards with characteristic mass labels. With the help of this glycan retention Time Grid (glyco-TiGr), individual chromatographic runs—some with strongly deviating conditions—could be projected to a model chromatogram with deci-minute precision. Experimental retention times are thereby converted to “virtual minutes” (vi-min) that are used much in the sense of the well-tried “glucose units” known from hydrophilic interaction chromatography^8,9^ and capillary gel electrophoresis.^37,38^ The developed approach revealed yet undescribed structures with bisecting Lewis-X in the human brain *N*-glycome.

## Experimental Section

### Materials

For the preparation of reference glycans from simple scaffolds, recombinant glycosyltransferases were expressed in the baculovirus insect cell system or purchased as detailed in the Supporting Information. Human brain samples were kindly provided by Dr. Lena Hirtler (Medical University, Vienna).

### Glycan Preparations

**Figure 2:**
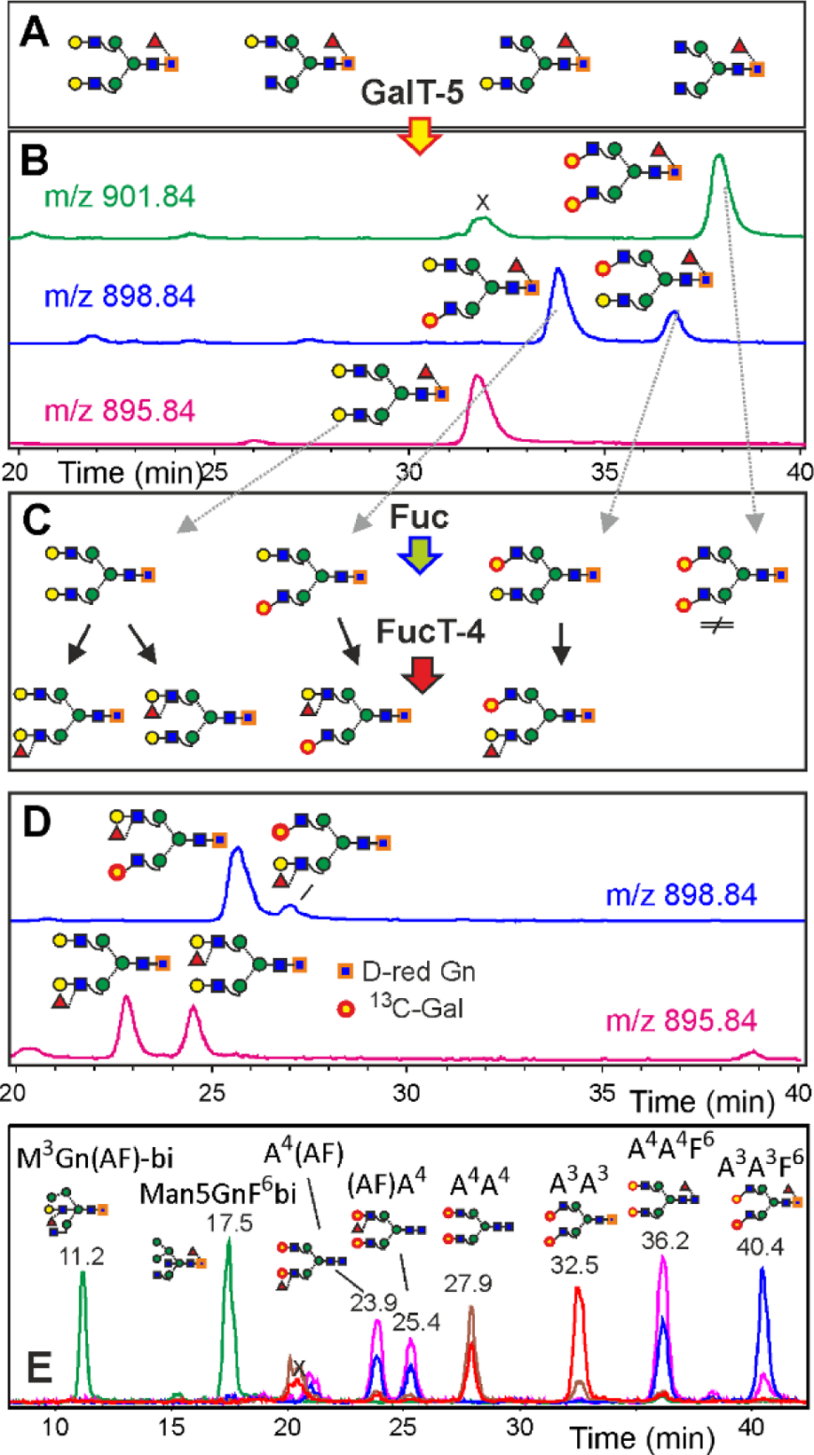
Simultaneous determination of retention times by isomer specific isotope labeling and depiction of the retention time grid. Neutral human IgG *N*-glycans (A) were deuterium-reduced and β1,3-galactosylated by βGalT5 with UDP-^13^C_6_-galactose. The products were analyzed by PGC-LC-ESI-MS (B) and treated with fucosidase and FucT-4 (C) to generate the four LeX isomers (D). Panel E shows the EICs for the doubly charged ions of eight isotope-labeled internal reference glycans used for retention time normalization, whereby green, magenta, brown, red, and blue are for *m*/*z* = 895.9, 901.4, 828.4, 901.4, and 901.9, respectively.

*N*-Glycans from immunoglobulins, bovine fibrin, white beans, and brain were prepared using PNGase F as described.^39,40^ Glycans were reduced with either NaBH_4_ or NaBD_4_ and eventually fractionated by PGC-LC monitored by MALDI-TOF MS.^39^ These glycans were used as scaffolds for the glycosyltransferases and glycosidases mentioned above for the preparation of the structures depicted in Figure 1. Some modifications were performed with UDP-^13^C_6_-galactose (Figure 2). Details of the preparation procedures are provided in the Supporting Information.

### Liquid Chromatography–Electrospray Ionization–Mass Spectrometry

Analytical PGC-LC-ESI-CID-MS/MS was performed with a capillary column (Hypercarb, 100 mm × 0.32 mm, 5 μm particle size; Thermo Scientific, Waltham, MA, USA) eluted with 10 mM ammonium bicarbonate as the aqueous solvent.^17,41^ Details of the preparation procedures are provided in the Supporting Information.

## Results

Thirty-six isomers of the fundamental composition H5N4F1 that may naturally occur in mammals plus four isomers eventually cropping up in glyco-engineered plants were biosynthetized from scaffold glycans by the use of recombinant glycosyltransferases (Figure S1). The choice of this type of composition was guided by reports on the role of fucosylation for synapsin expression^42^ and on the prevalence of other isomeric structures than the standard biantennary core fucosylated oligosaccharides, such as found on serum proteins like IgG, in brain glycoproteins.^22,30,43,44^

The many isomeric structures (Figure 1) need to be named, and we hope to find the reader inclined to accept the system applied herein, which allows to fully define structures with short text strings, naming the terminal residues as explained in ref (36) and in the Supporting Information.

### Preparation of Standards I—Individual Structures

The biosynthesis of biantennary glycans either with core fucose, Lewis X (LeX), or Lewis A (LeA) determinants or with the blood group H (bgH) α1,2-fucose started from isolated GnGnF^6^, A^4^GnF^6^, GnA^4^F^6^, and A^4^A^4^F^6^ peaks from human IgG.^40^ The structures are depicted in Figure 1 with consecutive numbers. One route entailed immediate β1,3-galactosylation, yielding the core-fucose-series (structures 1–4). De-fucosylation of these standards resulted in biantennary glycans that were individually treated with either FucT-III, which is primarily an α1,4-fucosyltransferase (structures 9–12), FucT-IV, to form LeX epitopes (5–8), or FucT-II, which leads to bgH type glycans (structures 13–20) (Figure S1). Another fate of the core-fucosylated glycans was to serve as scaffolds for the synthesis of α-galactosylated biantennary structures (structures 32–37). A large set of hybrid-type structures was synthesized from Man5 via Man5Gn. A complex variety of reactions then led to hybrid-type glycans with core or outer arm fucoses with surprising results, as detailed in the following text (structures 21–31). The products, when subjected to PGC-LC-ESI-MS, demonstrated the impressive selectivity of the PGC stationary phase with retention times spanning a 30 min window (Figures 2, S2).

### Preparation of Standards II—Differentially Isotope-Labeled Isomer Ensembles

With the aim of providing a concise retention time library, we considered stable isotope labeling for generating a series of reference glycans on the one hand and internal standards on the other hand. Deuterium introduction at the reducing end, galactosylation with one or two residues of ^13^C_6_-galactose, or use of ^13^C_2_–N-acetylated compounds allowed for mass increases of 1, 6, and 8 Da and in combination a panel of well over 10 different increments. Careful choice of the preparation scheme equipped many of the standards with individual mass labels, thus allowing unambiguous identification of otherwise isobaric compounds in one chromatographic analysis. A scheme for biosynthesis of a set of isomers with inherently different mass increments and the resulting extract ion chromatograms (EICs) are depicted for core fucosylated and LeX isomers (Figure 2) and for LeA and bgH isomers (Figures S3 and S4). The key point was the use of a mixture of A^4^A^4^, A^4^A^3^, A^3^A^4^, and A^3^A^3^—derived from human IgG as described above—with ^13^C_6_ galactose as the 3-linked terminal hexose, thereby introducing three mass levels for four (or eight in the case of the bgH series) isomers. These were combined with NaBH_4_ reduction (Lewis A series), NaBD_4_ reduction (both Lewis X and Core series as they elute far apart), and ^13^C_2_ acetyl groups (bgH series). Within these mixtures, isobaric core fucose, Lewis X, or Lewis A structures could be assigned via the individual standards (see above), the marked difference in abundance of 3- and 6-arm galactose in human IgG,^45^ and the pronounced fragmentation of the 3-arm one in positive mode CID (Figures S5 and S6). In the case of the bgH-series, the bias of the α1,2-FucT for type I chains, together with single standards, likewise allowed for unambiguous assignment of all peaks (Figure S4).

**Figure 3:**
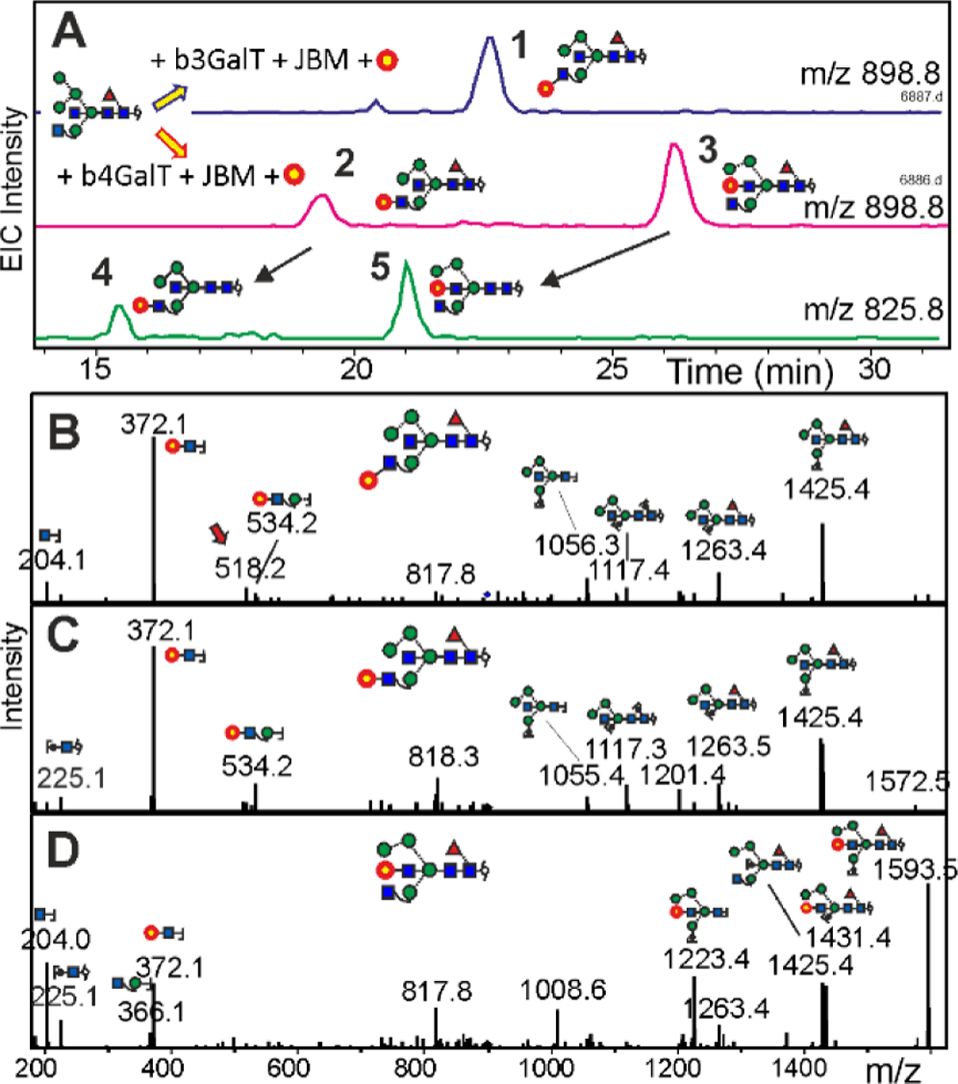
Evidence for the formation of hybrid-type *N*-glycans with bisecting LacNAc. Panel A demonstrates the effect of ^13^C_6_-galactose transfer to Man5GnF^6^bi (BD_4_-reduced) by either β3GalT or β4GalT, followed by mild mannosidase digestion (compounds **1**, **2**, and **3**) and removal of the core fucose (peaks **4** and **5**). Peaks **3** and **5** coelute with peaks **541c** and **540b**, respectively (Figure 4). Panels B to D show the positive mode CID spectra of peaks **1**, **2**, and **3** (MH^+^ = 1796.68). Cartoons are only given for some y and b ions with tentative selection of possible isomers. The red arrow denotes a product of internal re-arrangement.

Preparation of hybrid-type glycans starting from Man5Gn led to Man5GnF^6^bi (structure 30). This compound was treated with β3GalT and β4GalT, and ^13^C_6_-galactose was expected to generate one compound each with Gal on the 3-arm. This naive assumption, however, had to be revised as β4GalT generated—in very different ratios—two products. Comparison of positive mode CID spectra revealed a similar architecture of two products (Figure 3), whereas the late eluting β4GalT product showed a characteristic, preferential loss of GlcNAc (Figure 3). Negative ion CID substantiated the view that in this third peak, galactose was bound to the bisecting GlcNAc (structure 27; M^3^GnF^6^A-bi) (Figure S7). For conversions with FucT-2 (resulting in structure 24; M^3^GnF^2-4^-bi) and FucT-4 (resulting in structure 31; M^3^Gn(AF)-bi), therefore, these three possibilities had to be considered (Figure S8). FucT-3 did not act on the galactosylated hybrid-type glycan, which is in line with a recent report on the suppressive effect of bisecting GlcNAc.^31^ The Le X elaboration of the 3-arm (structure 26) likewise did not work out.

A final pettiness was the definition of the structure of the 6-arm in the case of Man4 glycans. The anachronistic uncertainty about the structure of Man4 glycans was probed with α1,6-specific glycosidase (Figure S9). In agreement with mechanistic studies,^46^ the α1,6-linked mannose is preferentially cleaved, which leads to a pronounced forward shift on PGC. This was observed for all Man4 isomers in this study, and therefore, the 6-arm of all Man4 structures is defined as “M^3^”.^36^

### Preparation of Standards III—The Time Grid Concept

To make a glycan retention times library useful for PGC-LC, individual runs must undergo a process akin to image warping in 2D-electrophoresis. Experimental data are projected to a model chromatogram. The “glucose unit” method, popular for fluorescence-labeled glycans,^8,9^ was recently adopted for PGC-LC.^47^ However, in our hands, ionization efficiency and peak shape of the isomaltose oligosaccharides of eight and more glucose units failed to meet the required quality. Besides, the likelihood of differing sorption isotherms for compounds without and with amide functions makes these simple sugars doubtful for a stationary phase as delicate as PGC. We therefore decided for a set of eight isotope-labeled *N*-glycans with elution times covering the entire time range of the isomers in focus (Figure 2). This multipoint retention **Ti**me **Gr**id (glyco-TiGr) allowed us to correct for elution time shifts, so that real elution times obtained in a particular run could be converted to virtual times (virtual minutes = vi-min) in an arbitrarily defined model chromatogram. Interpolation of sample retention times between “glyco-TiGr” mix “sign posts”—facilitated by a dedicated Excel sheet (Supporting Information)—generates a list of normalized retention times that can be looked up in the retention time library (Table 1). Notably, while applied to the composition H5N4F1 in this study, the very same “TiGr mix” can also be used to express elution of glycans with other masses in a manner essentially independent of the individual column and gradient conditions as demonstrated by the accidental collection of glycans with compositions other than H5N4F1 (Table S1). The criteria for choosing the TiGr mix structures were their distribution over the elution range of the compounds of interest and their accessibility. More TiGr standards will eventually be added to cover the elution regions of larger and sialylated *N*-glycans.

**Table 1:** Retention Times of H5N4F1 Isomers in the Virtual Model Chromatogram^a^

#	proglycan code	ret. time (vi-min)	#	proglycan code	ret. time (vi-min)	#	proglycan code	ret. time (vi-min)
31	**M**^**3**^**Gn(AF)-bi**	**11.2**	41	A^3^A^3^F^3^	24.0	12	(FA)A^3^	34.5
21	M^3^A^4^F^6^bi	16.6	6	(AF)A^4^	25.4	16	F^2–3^A^4^	35.6
30	Man5GnF^6^bi	17.5	36	Gn(A^3–4^F^3^)	26.2	15	A^4^F^2–3^	36.2
22	M^3^F^2–4^bi	18.5	8	(AF)A^3^	26.5	1	A^4^A^4^F^6^	36.2
25	M^3^A^3^F^6^bi	18.8		⟨A^4^A^4^⟩	⟨27.9⟩	18	F^2–3^A^3^	36.8
38	A^4^A^4^F^3^	19.6	**7**	**A**^**3**^(AF)	27.9	17	A^3^F^2–3^	37.2
39	A^4^A^3^F^3^	19.8	9	A^4^(FA)	27.9	2	A^4^A^3^F^6^	37.6
24	**M**^**3**^**GnF**^**2–4**^**-bi**	21.0	11	A^3^(FA)	31.2	19	A^3^F^2–4^	37.8
23	M^3^F^2–3^bi	21.2	14	F^2–4^A^4^	31.6	33	A^3–4^GnF^6^	38.3
29	Man5(AnF)	21.8	28	Man5An^4^F^6^	32.4	3	A^3^A^4^F^6^	39.6
27	**M**^**3**^**GnF**^**6**^**A-bi**	22.7		⟨A^3^A^3^>	⟨32.5⟩	4	A^3^A^3^F^6^	40.4
37	(A^3–4^F^3^)Gn	23.5	10	(FA)A^4^	32.5	34	GnA^3–4^F^6^	41.1
5	A^4^(AF)	23.9	20	F^2–4^A^3^	32.9	35	A^3–3^GnF^6^	42.5
40	A^3^A^4^F^3^	24.0	13	A^4^F^2–4^	34.1	36	GnA^3–3^F^6^	44.1

a Structure codes can be deciphered by reference to Figure 1, by use of the proglycan084 applet or reading the explanation in the Supporting Information. To allow for facile referencing to Figure 1, the consecutive numbers (#) are added. Two anisobaric TiGr-reference glycans are marked by “⟨ ⟩”. Bold print denotes structures not described so far to the best of the authors knowledge. Retention times are given as virtual minute (vi-min) of the reference chromatogram.

Admittedly, not all of the 40 isomers are satisfactorily separated. However, some notoriously difficult and usually ignored questions can be answered at first sight without consultation of negative mode MS/MS, which helps to define type^48^ and location of outer arm fucosylation^12,49^ and provide a sufficient quality of the fragment spectrum. Linkage isomers are, however, easily distinguished by retention time, as seen at the example of IgA *N*-glycans, which contain some β1,3-galactose (Figure S10, Table S2).

### Application to Brain *N*-Glycans

**Figure 4:**
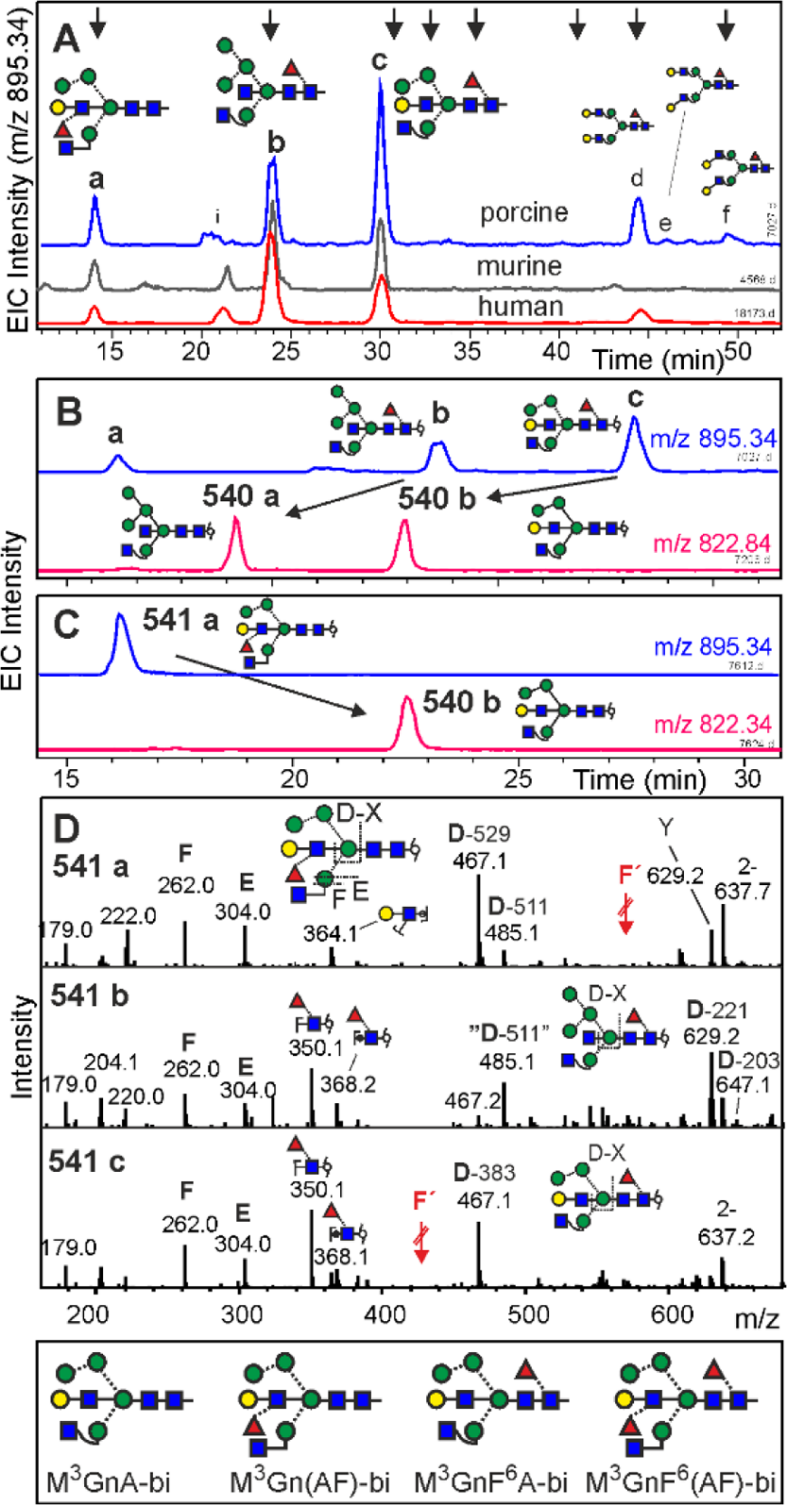
Analysis of brain *N*-glycans of H5N4F1 composition by PGC-LC-ESI-MS/MS. Panel A shows the EIC traces for brains from the pig, mouse, and human (from top to bottom). Arrows at the top indicate elution positions of the glyco-TiGr standard mix. Panel B illustrates the effect of bovine kidney fucosidase on pig brain glycans and panel C does so for the effect of α3/4-specific fucosidase on isolated peak **541a**. The negative ion CID spectra of the three peaks (panel D) all contain E- and F-ions arising from an unsubstituted 3-arm.^49^ Red arrows depict the positions of F-ions in the case of the relevant decorations. D-ions in the established sense would include the bisecting GlcNAc.^49^ The masses of the ejected bisecting groups of D-ions were calculated after resolving the structure. Fragment Y in 541a may hint at a co-existing Man5 structure, for which, however, reasoning about structures and elution times leaves hardly any possibility for this. The box at the bottom displays the structures of four brain *N*-glycan peaks containing bisecting LacNAc. Positive mode CID spectra are shown as Supporting Information S11.

Using the four series of biantennary standards (core, LeX, LeA, and bgH), we set out to compare their retention times with that found in the mouse brain. None of the 20 isomers coeluted with the three main peaks (Figure 4). The early elution time rather pointed at hybrid-type glycans, and in fact, peak **541b** coeluted with the reference.

Man5GnF^6^bi (structure 30) is a compound previously found in the brain.^30^ Galactosylated derivatives of this reference compound coeluted with brain peaks **541a** and **541c** and the galactose was bona fide imagined as linked to the 3-arm GlcNAc.

This could have been the end of the story, if we would not have been sensitized by the occurrence of two products in the galactose incorporation experiment with βGalT4 (Figure 3). In fact, brain peak **541c** perfectly coeluted with product **3** and was insensitive to galactosidase, as previously observed for bisecting LacNAc.^50^ Likewise, the structure exhibited the informative preferential loss of one terminal GlcNAc in positive mode CID (Figure S11) and the matching E- and F-type ions^49^ in CID of negative ions (Figure 4). Thus, peak 3 was identified as bearing a bisecting LacNAc (structure 27; M^3^GnF^6^A-bi). The isomer with a substituted 3-arm was not observed.

Making use of the very distinct elution positions of glycans with galactose on the 3-arm versus on bisecting GlcNAc (Figure 3a), we probed brain peak **541a** with a fucosidase able to act on the Lewis terminus. This converted peak **541a** to a compound coeluting with glycan **541b** (Figures 3a and 4c). Peak 1 furthermore gave a positive mode MS spectrum with a preferential loss of one GlcNAc as typical for a nonsubstituted 3-arm GlcNAc (Figure 4d). Thus, peak 1 harbored a glycan termed M^3^Gn(AF)-bi, in which the bisecting GlcNAc was fully elaborated to a Lewis X trisaccharide (structure 31; M^3^Gn(AF)-bi), whereas the corresponding isomer M^3^(AF)bi could not be found.

A rather large, single peak of composition H5N4F2 (15.0 vi-min) was converted to a peak akin to **541a** upon core-defucosylation and finally to M^3^GnA-bi (peak **540b**) upon complete defucosylation and thus was assigned the structure shown in Figure 4. The porcine sample additionally contained diantennary *N*-glycans that very likely originated from contaminating blood.

### Time Grid-Based Annotation Hyphenated to CID MS/MS

Support of assignments by CID spectra—manually or software driven—enhances the reliability of identification. With most options already ruled out by retention time, application of the usually more sensitive positive mode MS may be considered. Despite a certain inclination for fucose migration^51^—as clearly demonstrated by CID of asymmetrically isotope-labeled glycan—positive ion CID spectra contain valuable information about the branch location of fucose (Figures S5 and S6) or on the presence of structural elements such as GalNAc–GlcNAc units (*m*/*z* = 407) or α-Gal containing antennae (*m*/*z* = 528) (Figure S12). Nevertheless, negative-mode CID MS/MS of glycans usually yields more informative spectra^22,34,48^ and avoids deception by gas-phase re-arrangements.^51^ The value of information provided by separation is demonstrated by the truncated D-ions arising from bisected structures, which have lost all substituents of the β-mannose except that on the 6-arm. Substituents on the bisecting GlcNAc can only be guessed from failure of D and E/F ions to account for a glycans total mass, whereas their effect on retention is pronounced.

## Discussion

The selectivity achieved for the 40 isobaric *N*-glycans containing one fucose, five hexoses, and four *N*-acetylhexosamine residues almost overshot our expectations. In order to secure this treasure, normalization of retention times was realized by a set of synthetic stable isotope-labeled *N*-glycans spanning the entire oligosaccharide elution range. Obviously, these glyco-TiGr standards can also be applied to *N*-glycans with compositions other than H5N4F1 (Table 1). Likewise, the approach can be easily extended to larger, later eluting glycans. Population of further mass levels with a near to comprehensive collection of possible isomers would obviously result in an unparalleled isomer assignment power. Searched against a properly well-sorted virtual retention time library, the vi-min value of a peak at least excludes most of the possible isomers. Consultation of CID spectra then merely would help to confirm an assignment or choose between the very few remaining options. Vice versa, ambiguity of CID data can be met by information from chromatography. Notably, establishing libraries for just a few mass levels will allow us to infer the structures of glycans of other masses, as demonstrated here for the H5N4F2 peak. The hitherto, often covertly eluded distinction between different types of outer arm fucosylation or of β1,3 and β1,4 galactose (Supporting Information S2) becomes an easy task with the glyco-TiGr approach. Even the highly important but all too often neglected definition of the exact type of outer arm fucosylation becomes possible by just a one-shot analysis without the need for exo-glycosidase digestions and re-analysis.

The focus of the current work on the H5N4F1 composition level revealed the potential of the approach introduced herein and surfaced several hitherto undescribed structures for multimeric human IgA but foremost for brain glycoproteins.

Three prominent H5N4F1 *N*-glycan isomers were found in brains of mouse, pig, and humans. Most remarkably, one exhibited a bisecting LacNAc unit, that is, galactosylated bisecting GlcNAc or even a bisecting LeX unit. β4GalT, but not β3GalT5, was able to generate this exotic feature. Bisecting LacNAc has been found as a minor component in IgG.^49,50^ Bisecting LeX has been observed in a cell line deficient of GlcNAc transferase II.^52^ The exact structures of brain H5N4F1 peaks **a** and **c** have—to the best of the authors’ knowledge—not been reported so far. With a cumulated abundance in the several percent range (Figure S13) of the neutral *N*-glycans, which clearly predominate in the brain,^22,28^ bisecting LacNAc cannot be discounted as a curious trace component. The restriction to a few selected isomers raises questions about their biosynthesis of the rather special selection. The decoration of the bisecting GlcNAc in human brain glycans may be of particular significance because GnT-III, which produces the bisecting structures, shows altered expression levels in Alzheimer’s disease patients^53,54^ and other diseases.^55^ Therefore, interest in this element and other glyco-features experiences a renaissance.^22,56,57^

## Conclusions

The concept laid out herein could be the starting point for a full exploitation of the outstanding shape selectivity of porous graphitic carbon and thus for a methodology that truly appreciates the amazing and probably functionally significant isomeric diversity of *N*-glycans. Likewise, this study spotlights the brain *N*-glycome as a structurally unexplored territory, with broad implications for future deep structural glycomic studies on brain disorders such as Alzheimer’s disease.

## Data Availability

The data supporting the results of this study is available within the article and its Supporting Information files. Access to MS raw data files will be provided upon request.

## Supporting Information Available

Supporting methods, Tables with vi-min values of standards and IgA glycans, Figures exemplifying pathways for biosynthesis, examples of elution patterns of standards, positive and negative mode MS/MS spectra of standards, an example of hybrid-type isomers, determination of the Man4Gn structure, analysis of multimeric IgA glycans, MALDI-TOF MS of brain *N*-glycans, and explanation of the proglycan code (PDF)
Excel sheet for “vi-min” calculations (XLSX)
